# Intranuclear Delivery of HIF-1α-TMD Alleviates EAE *via* Functional Conversion of TH17 Cells

**DOI:** 10.3389/fimmu.2021.741938

**Published:** 2021-10-21

**Authors:** Jin-Su Shin, Ilkoo Kim, Jae-Seung Moon, Chun-Chang Ho, Min-Sun Choi, Sankar Ghosh, Sang-Kyou Lee

**Affiliations:** ^1^ Department of Biotechnology, Yonsei University College of Life Science and Biotechnology, Seoul, South Korea; ^2^ Department of Microbiology and Immunology, College of Physicians and Surgeons, Columbia University, New York, NY, United States; ^3^ Research Institute for Precision Immune-Medicine, Good T Cells, Inc., Seoul, South Korea

**Keywords:** hypoxia-inducible factor-1, Th17 cells, functional conversion, experimental autoimmune encephalomyelitis, protein transduction domain

## Abstract

T helper 17 (TH17) cells are involved in several autoimmune diseases such as multiple sclerosis (MS) and rheumatoid arthritis (RA). In addition to retinoic acid receptor-related orphan nuclear receptor gamma t (ROR-γt), hypoxia-inducible factor-1α (HIF-1α) is essential for the differentiation and inflammatory function of TH17 cells. To investigate the roles of HIF-1α in the functional regulation of TH17 cells under the normal physiological condition without genetic modification, the nucleus-transducible form of transcription modulation domain (TMD) of HIF-1α (ntHIF-1α-TMD) was generated by conjugating HIF-1α-TMD to Hph-1 protein transduction domain (PTD). ntHIF-1α-TMD was effectively delivered into the nucleus of T cells without cellular cytotoxicity. ntHIF-1α-TMD significantly blocked the differentiation of naïve T cells into TH17 cells in a dose-dependent manner *via* IL-17A and ROR-γt expression inhibition. However, T-cell activation events such as induction of CD69, CD25, and IL-2 and the differentiation potential of naïve T cells into TH1, TH2, or Treg cells were not affected by ntHIF-1α-TMD. Interestingly, TH17 cells differentiated from naïve T cells in the presence of ntHIF-1α-TMD showed a substantial level of suppressive activity toward the activated T cells, and the increase of Foxp3 and IL-10 expression was detected in these TH17 cells. When mRNA expression pattern was compared between TH17 cells and ntHIF-1α-TMD-treated TH17 cells, the expression of the genes involved in the differentiation and functions of TH17 cells was downregulated, and that of the genes necessary for immune-suppressive functions of Treg cells was upregulated. When the mice with experimental autoimmune encephalomyelitis (EAE) were treated with ntHIF-1α-TMD with anti-IL-17A mAb as a positive control, the therapeutic efficacy of ntHIF-1α-TMD *in vivo* was comparable with that of anti-IL-17A mAb, and ntHIF-1α-TMD-mediated therapeutic effect was contributed by the functional conversion of TH17 cells into immune-suppressive T cells. The results in this study demonstrate that ntHIF-1α-TMD can be a new therapeutic reagent for the treatment of various autoimmune diseases in which TH17 cells are dominant and pathogenic T cells.

## Introduction

T cell is a crucial component in the immune system, which orchestrates the various immune responses to infectious agents from outside of a body or danger signals from inside. However, the failure of immune regulation or genetic predisposition can induce the misdirected immune responses, which results in autoimmunity (1, 2). Especially, T helper 17 (TH17) cells, one of the helper T-cell subsets, are known to be related to the pathogenesis of several autoimmune diseases, and regulatory T cells (Treg cells) are the essential components of immune suppression (3, 4). Because overreactive TH17 cells and malfunction of regulatory T cells (Treg cells) are easily observed in inflammatory diseases, keeping the balance between TH17 and Treg cells is essential to prevent the autoimmune disease associated with chronic inflammation (5–7). It has been actively attempted to find the factors regulating the balance between TH17 and Treg cells to overcome autoimmunity. Transforming growth factor-β (TGF-β) could induce Treg cells decreasing the proportion of TH17 cells in inflammatory bowel disease mouse model, and oncostatin M could suppress the functions of TH17 cells in a rheumatoid arthritis mouse model *via* activation of Treg cells (8, 9).

TH17 cells are known to secret interleukin-17 (IL-17) and IL-23, and their differentiation can be inhibited by interferon-γ or IL-4, the signature cytokines of TH1 and TH2 cells, respectively. IL-17 and IL-23 act as proinflammatory cytokines, inducing inflammation in a body to remove foreign antigens. However, they can be related to autoimmunity as well in the presence of excess amount (10–12). The pathogenesis of some autoimmune diseases such as collagen-induced arthritis (CIA), inflammatory bowel disease (IBD), or psoriasis is caused by overactivated TH17 cells (13). For naïve T cells to differentiate into the TH17 subset, external signals such as TGF-β and IL-6 are required, which elevates the expression of *Rorc* gene encoding the signature transcription factor of the TH17 subset (14). Additionally, the expression of hypoxia-inducible factor-1α (HIF-1α) can be induced by TGF-β and IL-6 in many cell types and plays an essential role in the differentiation and function of TH17 cells (15, 16). IL-6 signaling induces HIF-1α expression, and TGF-β is known to stabilize HIF-1α proteins by inhibition of prolyl hydroxylase-2, which accelerates HIF-1α degradation *via* prolyl hydroxylation and protein ubiquitination (17). The stabilized HIF-1α protein forms HIF-1 complex together with HIF-1β, which acts as an essential transcription factor for Rorc and Il17a expressions. Because the other subunit of HIF-1 complex, HIF-1β, is constitutively expressed and the function of HIF-1β is limited to deliver HIF-1α into the nucleus (18, 19), HIF-1α expression by IL-6 signaling is critical for enhancing TH17 axis over Treg cells in the presence of TGF-β, the common cytokine in TH17 and Treg-cell differentiation. HIF-1α subunit also binds to Forkhead box P3 (Foxp3) protein and accelerates its degradation (20).

To modulate the differentiation or function of TH17 cells in the inflammatory microenvironment without genetic alteration, we used the novel strategy to inhibit the interaction of HIF-1α molecule with the promoter of HIF-1α-inducible genes by intranuclear delivery of TMD of HIF-1α using PTD. Previously, we identified the human-origin Hph-1-PTD and demonstrated that Hph-1-PTD could effectively deliver the cargo protein into the nucleus of the cells *in vitro* and *in vivo* (21–24). HIF-1α-TMD is composed of the DNA-binding domain and isotype-specific domain of HIF-1α. Nucleus-transducible form of HIF-1α-TMD (ntHIF-1α-TMD) is the fusion protein between HIF-1α-TMD and Hph-1-PTD which can be delivered into the nucleus and act as a competitive inhibitor by hijacking the binding partner of HIF-1α or occupation of hypoxia-response element (HRE) sequence of HIF-1α-inducible genes.

In this study, ntHIF-1α-TMD was effectively delivered into the nucleus of the cells in a dose- and time-dependent manner without any cellular cytotoxicity, and the activation of naïve T cells by TcR stimulation was not affected by ntHIF-1α-TMD treatment. Differentiation potential of naïve T cells into TH17 cells and their functions were inhibited by ntHIF-1α-TMD treatment *via* functional conversion of TH17 cells into immune-suppressive phenotype. Experimental autoimmune encephalomyelitis (EAE) symptoms were substantially alleviated by ntHIF-1α-TMD treatment. Therefore, ntHIF-1α-TMD can be an effective therapeutic reagent for the treatment of TH17-mediated autoimmunity.

## Materials and Methods

### Expression and Purification of ntHIF-1α-TMD

ntHIF-1α-TMD protein was expressed in *Escherichia coli* strain, BL21 Codon Plus (DE3) RIPL (Invitrogen, Waltham, MA, USA), and purified by affinity chromatography. In the purification step, cells expressing ntHIF-1α-TMD protein were harvested and sonicated with native lysis buffer (10 mM imidazole, 50 mM NaH_2_PO_4_, 300 mM NaCl). Inclusion bodies of centrifuged cell lysates were then sonicated with native lysis buffer containing 3 M urea. After centrifugation, supernatant was collected and ntHIF-1α-TMD was purified in native condition (wash buffer; 30 mM imidazole, 50 mM NaH_2_PO_4_, 300 mM NaCl, elution buffer; 500 mM imidazole, 50 mM NaH_2_PO_4_, 300 mM NaCl). Ion exchange chromatography was performed to remove endotoxin in the purified protein. Purified ntHIF-1α-TMD was desalted using PD-10 Sephadex G-25 column (GE Healthcare, Chicago, IL, USA) filled with 10% glycerol phosphate-buffered saline (PBS). The level of endotoxin in the fusion protein was approximately 6 EU/ml, which is within the safe range. When the fusion protein was injected into the mice, there was no sign of immune reaction by endotoxin such as anaphylactic shock by a cytokine storm.

### Immunoblot

Using sodium dodecyl sulfate polyacrylamide gel electrophoresis (SDS-PAGE), ntHIF-1α-TMD and HIF-1α-TMD were electrophoresed and blotted onto polyvinylidene fluoride (PVDF, Bio-Rad, Hercules, CA, USA). The membrane was blocked with a blocking buffer composed of 4% bovine serum albumin in Tris-buffered saline containing Tween 20 (TBST). The membrane was then incubated overnight at 4°C with anti-DYKDDDDK (Cell Signaling, Danvers, MA, USA). After TBST wash, TBST containing anti-mouse IgG (Abcam, Cambridge, MA, USA) was added to the membrane, and the membrane was incubated for 1 h at room temperature (RT). ECL reagent (Bio-Rad) was added onto the membrane, and the chemiluminescence signal from the membrane was detected using ChemiDoc (Bio-Rad).

### Cell Culture

Jurkat cells were cultured in RPMI-1640 medium (Lonza, Basel, Switzerland) containing 10% heat-inactivated fetal bovine serum (FBS, Hyclone, Logan, UT, USA), 2 mM l-glutamine (Lonza), and 100 U/ml penicillin/streptomycin (Lonza).

HeLa cells were cultured in DMEM medium (Lonza) containing 10% heat-inactivated fetal bovine serum (FBS, Hyclone), 2 mM l-glutamine (Lonza), 100 U/ml penicillin/streptomycin (Lonza), 1 mM sodium pyruvate, and 0.1 mM nonessential amino acids (NEAA, Lonza).

Mouse CD4+ T cells were cultured in RPMI-1640 medium (Lonza) containing 7.5% heat-inactivated fetal bovine serum (FBS, Hyclone), 2 mM l-glutamine (Lonza), 100 U/ml penicillin/streptomycin (Lonza), and 50 μM β-mercaptoethanol (Sigma-Aldrich, St. Louis, MO, USA). All cells were incubated at 37°C in a 5% CO_2_ environment.

### Protein Treatment

For protein delivery, 1 × 10^6^ cells of Jurkat cells on a 12-well cell culture plate (SPL) and 5 × 10^5^ cells of HeLa cells on a 4-well LabTek II chamber slide (Nunc, Roskilde, Denmark) were incubated with the fusion protein-containing media in the CO_2_ incubator for 1–2 h before analysis. After protein treatment, Jurkat cells were used in flow cytometry and HeLa cells were observed using a confocal microscope (LSM 880, Zeiss, Jena, Germany). Mouse naïve CD4+ T cells at 2 × 10^5^ were incubated with the fusion protein-containing media on a 96-well cell culture plate (Eppendorf) for 72 h under specific T-cell subset-skewing conditions.

### CD4+ T-Cell Differentiation

CD4+CD62L+ (naïve) T cells were purified using CD4+CD62L+ T-cell isolation kit II (Miltenyi Biotec, Bergisch Gladbach, Germany) in RBC-eliminated splenocytes from spleens of female C57BL/6 mice. The isolation step followed the protocol of the kit. After the isolation of CD4+CD62L+ T cells, they were cultured in a cell culture plate coated with 1 μg/ml anti-CD3ϵ (BD Bioscience, Franklin Lakes, NJ, USA) and anti-CD28 (BD Bioscience) for 3 days with or without proteins. Naïve CD4+ T cells were cultured with each cytokine mix for differentiation into TH1 (25 ng IL-12 and 2 μg/ml anti-IL-4), TH2 (200 ng/ml IL-4 with 2 μg/ml anti-IFN-γ), TH17 (1 ng/ml TGF-β, 25 ng/ml IL-6, 2 μg/ml anti-IL-4, and 2 μg/ml anti-IFN-γ), or induced Treg (iTreg, 5 ng/ml TGF-β with 20 ng/ml IL-2). Cytokines and antibodies were purchased from PeproTech (Rocky Hill, NJ, USA), R&D Systems (Minneapolis, MN, USA), or BioLegend (San Diego, CA, USA).

### Measurement of the Fusion Protein Localization

After protein treatment of HeLa cells on a four-well LabTek II chamber slide, the samples were washed with PBS and fixed with fixation solution (1% of methyl alcohol in formaldehyde) for 10 min at RT. Following PBS wash, the samples were treated with permeabilization solution (0.2% Triton X-100 in PBS) for 10 min at RT, and the remaining triton X-100 was removed by PBS wash. The samples were incubated with blocking solution (1% BSA in PBS) for 1 h at RT. Also, the delivered proteins were captured with anti-FLAG FITC (Sigma-Aldrich). After PBS wash, DAPI solution (Sigma-Aldrich) was treated for nuclear counterstaining. The samples were imaged with a confocal microscope (LSM 880, Zeiss).

### Flow Cytometry

Protein transduction efficiency in Jurkat cells was analyzed by intracellular staining with anti-DYKDDDDK PE (BioLegend) after a protein treatment.

The differentiated CD4+ T cells were stimulated with a cell-stimulation cocktail (eBioscience, San Diego, CA, USA) for 4 h. Their differentiation and function were analyzed by surface staining of each marker or intracellular staining of cytokines or transcription factors. Before intracellular staining, cells were fixed with IC fixation buffer or fixation/permeabilization buffer (eBioscience). Antibodies were purchased from eBioscience, BioLegend, or BD Bioscience.

Immune cells isolated from the lymph nodes or spinal cord of EAE-induced mice were stimulated with a cell-stimulation cocktail overnight before analysis. Their status was analyzed by staining with anti-CD4 APC, anti-IL-17A PE, or anti-Foxp3 FITC (eBioscience).

Flow cytometry was performed using FACSCalibur (BD Bioscience) or SA3800 (Sony, Tokyo, Japan), and the data were analyzed by the Flowjo V10 program.

### Reporter Gene Assay

The reagents for luciferase reporter gene assay were purchased from Promega (Madison, WI, USA), and the assay was conducted according to the manufacturer’s instructions. Using Bio-Rad Gene Pulser II, an electric pulse was applied to the mixture of Jurkat cells and IL-17 luciferase reporter plasmid with the equal amount of plasmid containing one of the receptor-related orphan receptor gamma t (ROR-γt), HIF-1α, or p300 gene with any combinations of them. After 24 h incubation, the cells were restimulated with PMA and ionomycin, and the luciferase activity in the cells was measured using GloMax 96 Microplate Luminometer (Promega).

### CCK-8 Assay

Sorted mouse naïve CD4+ T cells at 1 × 10^5^ were seeded into a 96-well plate (SPL) and treated with the fusion protein for 1 h. After protein treatment, cell counting kit-8 (Dojindo Laboratories, Kumamoto, Japan) reagent was added to the cultured cells. The cells were incubated at 37°C in a 5% CO_2_ environment for an additional 4 h to react to the reagent sufficiently. Because only viable cells could react to the reagents to make yellow-color dye, the viability of cells was measured by analyzing the absorbance of 450 nm wavelength using a microplate reader (Bio-Rad).

### Measurement of Cytokines

After 72 h incubation, the supernatant from the culture media of activated or differentiated CD4+ T cells was collected and analyzed by enzyme-linked immunosorbent assay (ELISA) for murine IL-2, IL-17A, or IL-10 according to the manufacturer’s protocol (eBioscience).

The blood was collected from the EAE mice at day 16 from disease induction, and the serum was analyzed by ELISA for murine IL-17A or IL-10. The procedure followed the manufacturer’s instructions (eBioscience).

The concentration of cytokines was measured by analyzing the absorbance of 450 nm wavelength using a microplate reader (Bio-Rad).

### Quantitative Real-Time PCR

Using RNeasy Mini Kit (Qiagen, Hilden, Germany) and a spectrophotometer (Thermo Scientific, Waltham, MA, USA), total RNA was extracted from CD4+ T cells and analyzed to confirm their concentration and integrity. cDNA was synthesized with isolated RNA using Maxime RT premix kit (iNtRON Biotechnology, Seongnam, South Korea). qRT-PCR was then performed with SYBR Premix Ex Taq (Takara, Kusatsu, Japan), ABI 3700, and real-time PCR system (Applied Biosystems, Waltham, MA, USA). Quantification of relative mRNA expression was normalized to endogenous β-actin. Primers were used to quantify the transcripts of *Rorc* gene: forward TGCAAGACTCATCGACAAGGC and reverse AGCTTTTCCACATGTTGGCTG.

### RNA-Seq and Gene Set Enrichment Analysis

Cultured mouse CD4+ T cells in TH17-skewing condition with or without ntHIF-1α-TMD were harvested at day 3. RNA extraction and sequencing were performed by Macrogen (Seoul, South Korea). By removing the low-quality data, adaptors, and contaminant DNA, original read data were trimmed using Trimmomatic. Trimmed read data were aligned using genomic reference (10 mm) using HISAT2, and transcripts per million (TPM) counts were acquired from the assembled transcript using StringTie.

A gene set enrichment analysis (GSEA) was performed on the ntHIF-1α-TMD-treated sample using TPM counts of differentially expressed genes compared with TH17 cells with an absolute value of fold change ≥2 and exactTest raw *p*-value <0.05. We used MSigDB, including immunesigDB (25) to analyze the immunologic signature gene sets.

### Suppression Assay

After 72 h incubation in TH17-skewing condition with or without the protein, T cells were harvested and washed with PBS. Freshly isolated CD4+CD62L+ T cells were labeled by cell proliferation dye (eBioscience) and cultured with differentiated T cells. The cells were plated in the presence of 0.5 μg/ml of anti-CD3ϵ (BD Bioscience) and 50 μg/ml of mitomycin-treated (Sigma-Aldrich) feeder cells (CD4-depleted splenocytes) in 96-well round-bottom plate (Corning, Corning, NY, USA). As a control, iTreg cells differentiated from naïve CD4+ T cells for 72 h were used to coculture with freshly isolated CD4+CD62L+ T cells treated as above. Flow cytometry was performed after 72 h incubation to analyze the suppression activity.

### EAE Induction and Scoring

Female C57BL/6 mice (6-week-old) were purchased from Orient Bio (South Korea) and housed in the specific pathogen-free facility of Yonsei Laboratory Animal Research Center (YLARC). Peptide (MEVGWYRSPFSRVVHLYRNGK) from amino acid residue 35–55 of myelin oligodendrocyte glycoprotein (MOG) was synthesized by Anygen (Daegu, South Korea). Mice were immunized with two subcutaneous injections of a total 200 μg of the MOG35-55 peptide emulsified in 200 μl of complete Freund’s adjuvants (Sigma-Aldrich) containing 200 μg of the heat-killed mycobacterium tuberculosis (BD Bioscience) on day 0. On days 0 and 2, 400 ng of pertussis toxin (List Biological, Campbell, CA, USA) was administered by intraperitoneal injection. The clinical score of mice was measured daily starting day 7. Scoring was as follows (26): 0, normal; 1, weakness of tail; 2, paralyzed tail; 3, weakness of hind limbs and uncoordinated movement; 4, paralyzed one hind limb; 5, paralyzed both hind limbs; 6, paralyzed both hind limbs and weakness of forelimbs; 7, paralyzed both hind limbs and one forelimb; 8, paralyzed both hind limbs and both forelimbs; and 9, death.

Experimental procedures were approved by the Institutional Animal Care and Use Committee (IACUC) of Yonsei University (South Korea), and all animal experiments were performed with the IACUC guidelines for the ethical use of animals (IACUC-201710-634-03, IACUC-201805-729-03).

### Histologic Studies

Isolated spinal cords from the fusion protein-treated mice or MOG-immunized mice were fixed by 4% phosphate-buffered paraformaldehyde. These samples were embedded in paraffin, and the paraffin sections were stained with H&E, Luxol fast blue (Diagnostic BioSystems, Pleasanton, CA, USA), and anti-CD4 (Abcam). Tissue staining and acquisition of images were performed by GENOSS (Suwon-si, South Korea). The images were acquired from the adjacent sections of the spinal cord, and CD4+ T cells were counted per section.

### Statistical Analysis

The results are expressed as a mean ± SEM (*n* ≥ 3). Statistical analysis of group differences was examined using an unpaired Student’s *t*-test. The number of asterisks indicated the following significance: ns; not significant, ^*^ *p* < 0.05, ^**^ *p* < 0.01, and ^***^ *p* < 0.001.

## Results

### ntHIF-1α-TMD Can Be Delivered Into the Nucleus of T Cells in a Dose- and Time-Dependent Manner

Induction of retinoic acid ROR-γt expression is essential for TH17-cell differentiation and functionality of TH17 cells. Through IL-6-STAT3 or mammalian target of rapamycin (mTOR) signaling, the activated HIF-1α induces ROR-γt expression, resulting in TH17-cell-related transcription (27). In T-cell-specific HIF-1α-knockout (KO) study, loss of intrinsic HIF-1α showed impaired TH17 function and elevated Foxp3 expression. However, in another HIF-1α-KO study, HIF-1α was an essential factor for suppressive activity of Treg in hypoxic conditions (27, 28). To verify the functional importance of HIF-1α in TH17 cell in the normal physiological state without genetic modification such as KO or overexpression of HIF-1, ntHIF-1α-TMD was generated as a competitive inhibitor of HIF-1α-mediated transcription, which contains Hph-1-PTD and N-terminal 245-amino acids of HIF-1α (**Supplementary Figure S1A**). This N-terminal domain of HIF-1α is known to have the essential amino acid sequences for its binding to hypoxia-response element (HRE) sequence and HIF-1β and isotype specificity (20). HIF-1α-TMD without Hph-1-PTD is used as a negative control. ntHIF-1α-TMD was expressed in the *E. coli* system and purified using Ni-NTA agarose. The identity of the purified ntHIF-1α-TMD or HIF-1α-TMD was confirmed by SDS-PAGE or Western blot using anti-FLAG (**Supplementary Figure S1B**). As shown in **Supplementary Figures S1C, D**, ntHIF-1α-TMD can be delivered effectively into the nucleus of Jurkat or primary T cells in a dose- and time-dependent manner when these T cells were incubated with ntHIF-1α-TMD for 2 h. The delivered ntHIF-1α-TMD remained stably inside the cells and peaked at 24 h and gradually declined until 96 h of postdelivery. To monitor the intracellular localization of the delivered proteins, HeLa cells were treated with ntHIF-1α-TMD, and the location of ntHIF-1α-TMD or nucleus was visualized by FITC-labeled anti-FLAG mAb and DAPI, respectively. The ntHIF-1α-TMD was detected inside the nucleus using confocal microscope after 2 h of incubation (**Supplementary Figure S1E**). To examine whether the treatment of T cells with ntHIF-1α-TMD exerts any cellular cytotoxicity, mouse naïve CD4+ T cells were treated with various concentrations of ntHIF-1α-TMD up to 3 µM. The intranuclear delivery of a high concentration of ntHIF-1α-TMD did not affect the cell viability (**Supplementary Figure S1F**). These results demonstrate that ntHIF-1α-TMD can be delivered into the nucleus of the cells effectively and stably without cellular cytotoxicity.

### ntHIF-1α-TMD Specifically Inhibits the Differentiation of Naïve T Cells Into TH17 Cells

**Figure 1:**
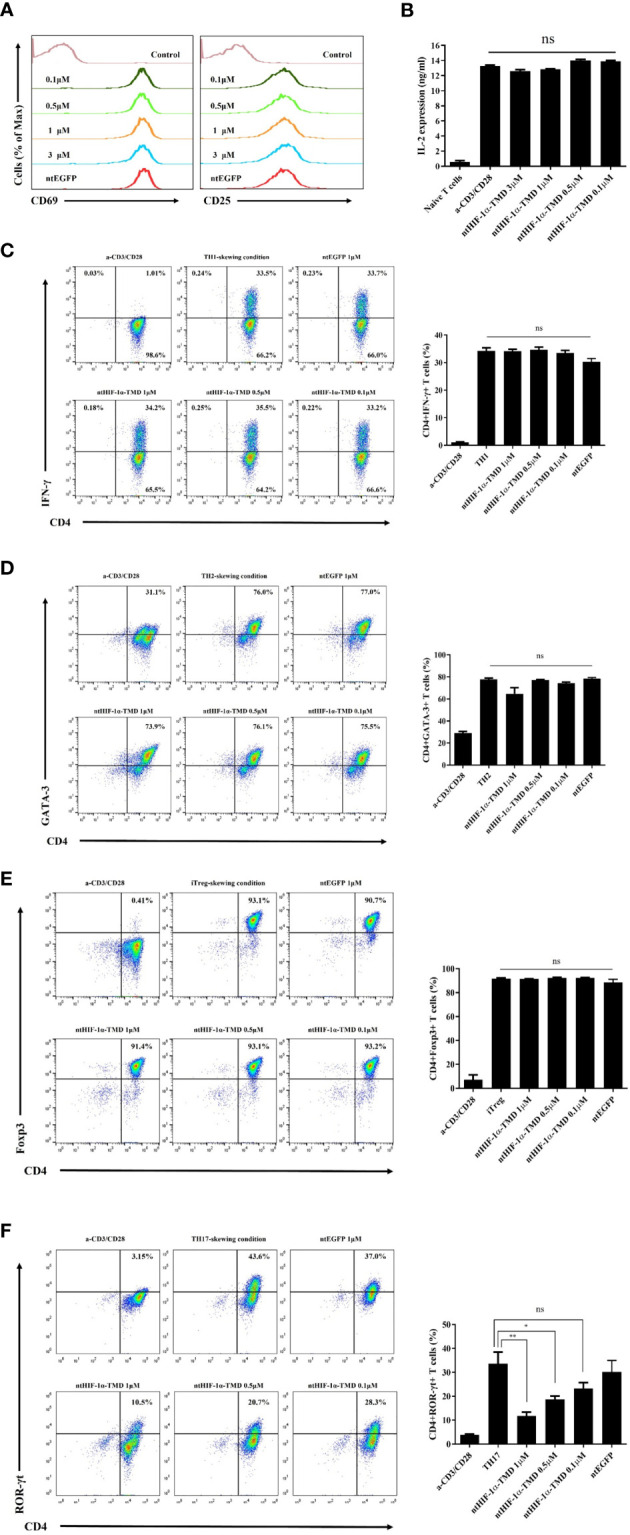
Examination of the functional influence of ntHIF-1α-TMD on CD4+ T-cell subsets. **(A)** The induced expression of the surface proteins associated with T-cell activation was analyzed by flow cytometry on CD4+ T cells activated by anti-CD3ϵ and anti-CD28 with or without ntHIF-1α-TMD treatment. Hph-1-PTD-fused enhanced green fluorescent protein (ntEGFP) was used as a negative control. **(B)** The secreted IL-2 in the supernatant from the cells in **(A)** was measured by ELISA. **(C**–**F)** The functional influence of ntHIF-1α-TMD on TH1, TH2, iTreg, or TH17 cells was evaluated by flow cytometry after 72 h incubation in the T-cell subset-skewing condition with ntHIF-1α-TMD. All experiments shown here were repeated three times. Data are represented as mean ± SEM (*n* ≥ 3), and the statistical analysis was examined using Student’s *t*-test. ns, not significant, *P < 0.05, **P < 0.01.

CD4+ T cells differentiate into the specialized subset in the pathogen-specific microenvironment to induce the proper immune responses against foreign pathogens. However, chronic activation of specific CD4+ T-cell subset could be a possible pathogenic factor for inflammatory diseases in a T-cell subset-dependent manner (29). To examine the functional importance of HIF-1α for the differentiation potential of CD4+-naive T cells into various CD4+ T-cell subsets, mouse naïve T cells were cultured in T-cell subset-polarized condition in the presence of ntHIF-1α-TMD. The generation of TH1, TH2, TH17, or Treg cells was evaluated by intracellular staining of T-cell subset-specific transcription factor or cytokine. Because T-cell receptor (TcR)-mediated T-cell activation is the common and initial signaling event for the differentiation of all of T-cell subsets, the functional influence of ntHIF-1α-TMD on T-cell activation events such as the induction of CD69 or CD25 on the surface and the secretion of IL-2 were examined. As shown in **Figures 1A, B**, ntHIF-1α-TMD treatment did not affect TcR-mediated early activation signaling events. While treatment of CD4+-naïve T cells with ntHIF-1α-TMD did not alter their differentiation capacity into TH1, TH2, or Treg cells, the differentiation program of CD4+-naïve T cells into TH17 cells was significantly impaired by ntHIF-1α-TMD (**Figures 1C–F**).

### Inhibition of TH17-Cell Differentiation by ntHIF-1α-TMD Is Attributed to Blockage of HIF-1α-Mediated Transcription

**Figure 2:**
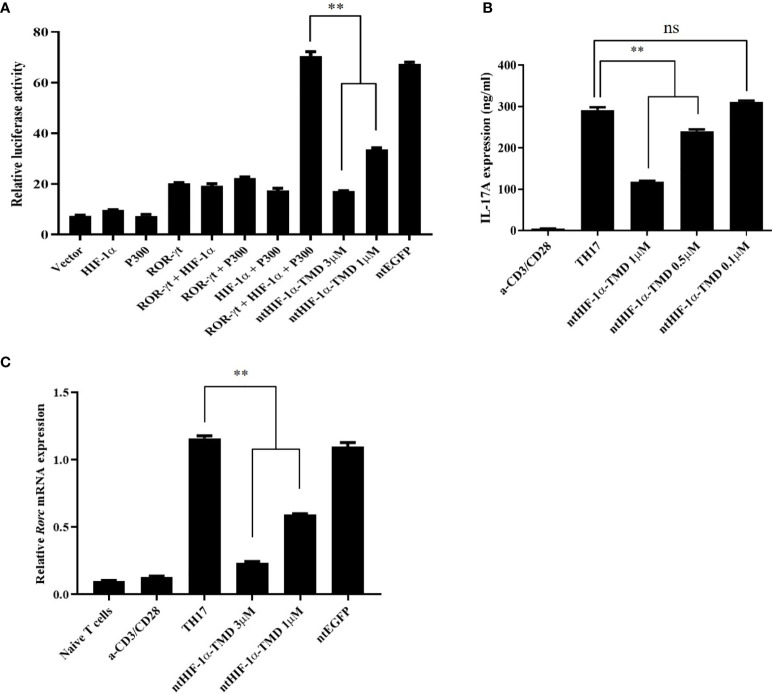
Inhibitory effect of ntHIF-1α-TMD on differentiation and function of TH17 cells. **(A)** Jurkat T cells were transfected with the plasmid containing the luciferase reporter gene whose expression is driven by the IL-17 promoter or the indicated gene. Luciferase activity was measured after ntHIF-1α-TMD treatment. **(B**, **C)** After incubation of naïve CD4+ T cells for 3 days in TH17-skewing condition, the level of secreted IL-17A in the supernatant was measured by ELISA. qRT-PCR was performed to evaluate the effect of ntHIF-1α-TMD on *Rorc* transcription. Experiments were performed in triplicated wells and repeated three times. Data are represented as mean ± SEM (*n* ≥ 4). Statistical analysis was performed using Student’s *t*-test. ns, not significant, **P < 0.01.

The expression of IL-17, a major inflammatory cytokine that TH17 cells secrete upon activation, is induced by the complex of transcription factors (ROR-γt/HIF-1α/p300). To validate whether the inhibitory function of ntHIF-1α-TMD in differentiation program of TH17 cells is mediated by HIF-1α-dependent transcription, the plasmid in which the expression of the luciferase reporter gene is driven by IL-17 promoter was transfected into Jurkat T cells together with the plasmid containing the gene encoding ROR-γt, HIF-1α, or p300. ROR-γt only, ROR-γt and HIF-1α, or HIF-1α and p300 enhanced IL-17 promoter-mediated transcription. However, the highest luciferase activity was observed in the group transfected with all three plasmids encoding ROR-γt or HIF-1α or p300. The induced-luciferase activity and IL-17 secretion from TH17 cells were significantly reduced by ntHIF-1α-TMD treatment in a dose-dependent manner (**Figures 2A, B** **)**. Next, we evaluated the level of *Rorc* mRNA and ROR-γt protein in TH17 cells differentiated from naïve CD4+ T cells in the presence of a different concentration of ntHIF-1α-TMD. As shown in **Figures 1F**, **2C**, the ntHIF-1α-TMD treatment effectively inhibited the transcription of *Rorc*, leading to the reduction of ROR-γt protein in a concentration-dependent manner. These results demonstrate that ntHIF-1α-TMD can specifically prevent the differentiation of CD4+-naïve T cells into TH17 cells and the function of TH17 cells through the competitive inhibition of the HIF-1α-mediated transcription.

### ntHIF-1α-TMD Can Induce the Functional Conversion of TH17 Cells Into the Immune-Suppressive T Cells

**Figure 3:**
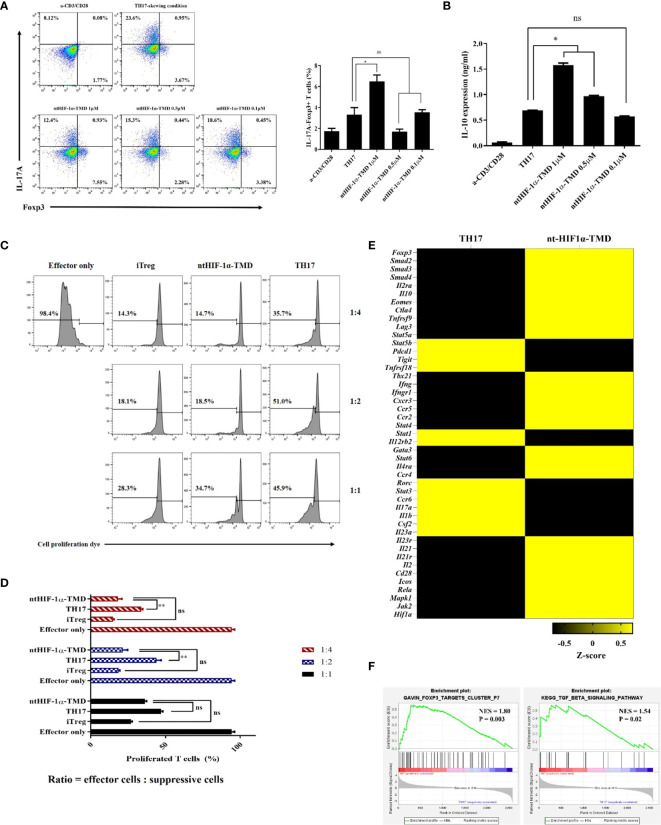
The functional conversion of TH17 cells into T cells with the immune-suppressive phenotype. **(A)** In the TH17-skewing condition, the change of IL-17A or Foxp3 expression in TH17 cells treated with ntHIF-1α-TMD were analyzed by flow cytometry. **(B)** The amount of IL-10 in the supernatant from T cells in **(A)** was measured by ELISA. **(C, D)** The immune-suppressive function of TH17 cells treated with ntHIF-1α-TMD was evaluated by suppression assay. **(E)** Expression heatmap of helper T cell-related genes. Z-score was calculated from log2(TPM+1). **(F)** GSEA-based enrichment plots of the Foxp3 targets cluster or TGF-β signaling pathway. Experiments in **(A**–**D)** were performed three times. Data are represented as mean ± SEM (*n* ≥ 3), and Student’s t*-*test was performed for statistical analysis. ns, not significant, *P < 0.05, **P < 0.01.

Functional plasticity or conversion of CD4+ T cell into specific T-cell subset plays a critical role in induction and progress of autoimmunity (30, 31). Previously, it was reported that HIF-1α accelerates the degradation of Foxp3 protein through the oxygen-dependent degradation (ODD) domain of HIF-1α in TH17-skewing condition (20). Therefore, we hypothesized that ntHIF-1α-TMD having a Foxp3-binding motif and no ODD domain might stabilize Foxp3 in TH17 cells. The level of Foxp3 and intracellular IL-17A was evaluated in TH17 cells when the differentiation of CD4+ T cells into TH17 cells was induced in the presence of different ntHIF-1α-TMD concentrations. ntHIF-1α-TMD treatment increased Foxp3 protein accompanied by the reduction of IL-17A in TH17 cells. The increase of Foxp3 protein substantially enhanced the secretion of immune-suppressive cytokine IL-10 into the culture medium (**Figures 3A, B** **)**. To examine whether TH17 cells functionally converted by ntHIF-1α-TMD treatment exert the immune-suppressive activity toward effector T cells, the suppression assay was performed with TH17 cells or ntHIF-1α-TMD-treated TH17 cells. iTreg cells were used as a positive control. As shown in **Figures 3C, D**, ntHIF-1α-TMD-treated TH17 cells showed a substantial level of suppressive potential to effector T cells comparable with iTreg cells. To investigate the change of mRNA expression profile induced by ntHIF-1α-TMD treatment, we prepared mRNA from TH17 cells or TH17 cells treated with ntHIF-1α-TMD and performed mRNA sequencing using Illumina platform. Based on normalized TPM counts, the expression pattern of the representative genes was compared between these two cell populations. The expression of the genes associated with differentiation and function of TH17 cells such as *Rorc*, *Stat3*, and *Il17a* were reduced, and the genes necessary for the immune-suppressive functions such as *Foxp3*, *Il10*, and *Smad3* were upregulated by ntHIF-1α-TMD treatment (**Figure 3E**). Furthermore, according to the gene set enrichment analysis (32, 33), the expression of Foxp3-targeting genes and the genes involved in the TGF-β signaling pathway was significantly upregulated by ntHIF-1α-TMD treatment (**Figure 3F**). Therefore, functional inhibition of the endogenous HIF-1α by ntHIF-1α-TMD can convert the inflammatory TH17 cells into T cells with immune-suppressive phenotype.

### ntHIF-1α-TMD Can Effectively Alleviate the Symptoms of EAE

**Figure 4:**
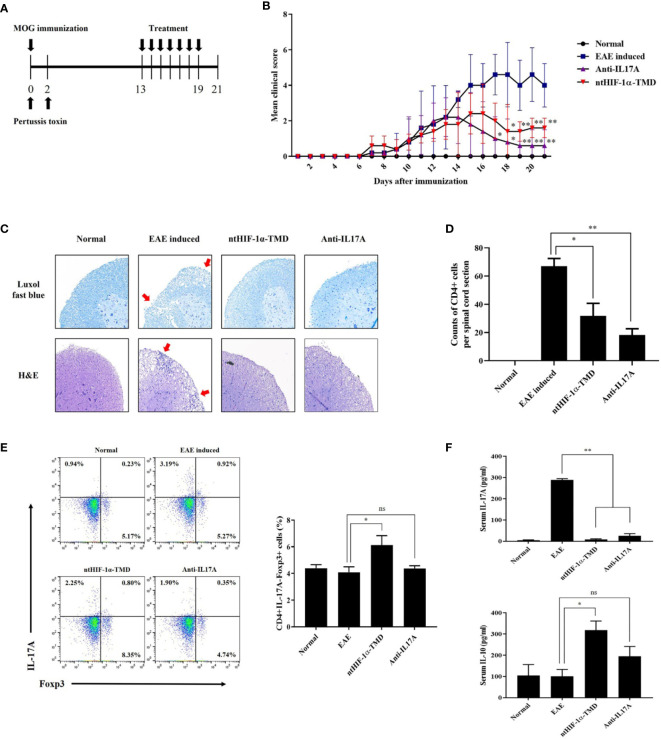
Therapeutic potential of ntHIF-1α-TMD in EAE disease models. **(A)** The treatment scheme of EAE-induced mice with phosphate-buffered saline (EAE induced), ntHIF-1α-TMD (100 μg/mouse), or anti-IL17A (40 μg/mouse) is represented. **(B)** A clinical score was measured until 21 days after EAE induction. **(C)** EAE-induced mice (w/or w/o treatment) were sacrificed 16 days after EAE induction. The spinal cords were harvested, and the histological analysis was performed by Luxol Fast Blue **(C**, upper panel**)** and H&E **(C**, lower panel**)** staining. **(D)** CD4+ T cells infiltrated into the spinal cord in **(C)** were counted, and the graph is represented as mean ± SEM (*n* ≥ 4). **(E)** CD4+ T cells in the lymph nodes harvested from the mice at day 16 were analyzed for the expression of IL-17A or Fopx3 by flow cytometry. **(F)** The level of IL-17A or IL-10 in the serum of mice at day 16 was measured by ELISA. The graphs are represented as mean ± SEM (*n* ≥ 3). The experiments in EAE disease model were independently performed three times. The group differences were analyzed by Student’s *t*-test as a statistical analysis. ns, not significant, *P < 0.05, **P < 0.01.

It has been reported that TH17 cells are the critical pathogenic player in multiple sclerosis (MS), rheumatoid arthritis (RA), and juvenile idiopathic arthritis (JIA) (34–36). To determine whether ntHIF-1α-TMD shows the therapeutic potential to the animal model with autoimmunity, the mice with EAE, animal model of multiple sclerosis (MS), were treated with ntHIF-1α-TMD as scheduled in **Figure 4A**. Anti-IL-17A mAb was used as a positive control. Compared with the PBS-treated group, ntHIF-1α-TMD- or anti-IL17A mAb-treated group showed a significant improvement in the clinical score and the level of demyelination in the spinal cord (**Figures 4B, C** **)**. When the number of CD4+ T cells infiltrating the spinal cord was examined, the significant reduction of CD4+ T cells in the spinal cord was observed in anti-IL-17A mAb- or ntHIF-1α-TMD-treated group (**Figure 4D**). To understand how T-cell populations are affected by ntHIF-1α-TMD treatment in EAE-induced mice, the immune cells were prepared from the lymph nodes, and the level of intracellular IL-17A or Foxp3 was examined. Although the number of CD4+IL-17A-Foxp3+ T cells increased in both anti-IL-17A mAb- and ntHIF-1α-TMD-treated groups, the population change in CD4+IL-17A+ T cells or CD4+IL-17A-Foxp3+ T cells was significant only in the ntHIF-1α-TMD-treated group (**Figure 4E** and **Supplementary Figure S2A**). Additionally, in spinal cord-infiltrated cells, CD4+IL-17A+ T cells were significantly reduced by ntHIF-1α-TMD treatment, and serum IL-10 was elevated only in the ntHIF-1α-TMD-treated group while the level of IL-17A in the serum decreased in both treatment groups (**Supplementary Figure S2B** and **Figure 4F**). Taken these results together, ntHIF-1α-TMD treatment can improve the symptoms of EAE *via* functional conversion of TH17 cells into T cells with the immune-suppressive phenotype.

## Discussion

Each subset of CD4+ T cells can be defined by expression of distinct transcription factors, T-bet in TH1, GATA-3 in TH2, ROR-γt in TH17, and Foxp3 in Treg. Beyond this classification, each CD4+ T-cell subset can demonstrate functional plasticity in a particular inflammatory environment. For example, IL-12 receptor signaling in TH17 cells enhances IFN-γ secretion, and IL-1β, IL-21, and IL-23 treatment induces the conversion of Treg cells into IL-17-producing cells (30, 37). The subset-specific transcription factors act as a critical regulator in the functional conversion of T cells and the autoimmune microenvironment.

HIF-1α is thought as the essential transcription factor for the cellular reaction to a hypoxic condition, and its function in immune cells, especially in TH17, is known to be related to IL-6 signaling and the mTOR pathway (27, 38). Knockout of HIF-1α in T cells resulted in decreased expression of ROR-γt and suppressed TH17 differentiation. In mice lacking HIF-1α in CD4+ T cells, EAE induction was suppressed because of decreased IL-17-producing cells and increased Foxp3+ Treg cells. However, the influence of HIF-1α deletion on functions of TH17 or Treg cells may differ depending on the cellular microenvironment (20, 27, 28).

In this study, we attempted the novel strategy to deliver TMD of HIF-1α into the nucleus of T cells using Hph-1-PTD to investigate the function of HIF-1α in normal physiological conditions without genetic alteration. ntHIF-1α-TMD, the nucleus-deliverable form of HIF-1α-TMD, can competitively inhibit the functions of the endogenous HIF-1α in T cells. ntHIF-1α-TMD can be delivered into the nucleus of T cells in a dose- and time-dependent fashion and remains inside the cells stably without any cellular cytotoxicity.

Intranuclear delivery of HIF-1α-TMD into naïve T cells during induction of TH17-cell differentiation significantly blocked TH17-cell differentiation by transcriptional inhibition of the genes involved in the functions of TH17 cells, such as IL-17A and ROR-γt. However, ntHIF-1α-TMD treatment of naïve T cells affects neither the differentiation potential into TH1, TH2, or Treg cells nor early and late signaling events for T-cell activation such as induced expression of CD69, CD25, or IL-2. Surprisingly, ntHIF-1α-TMD-treated TH17 cells acquired immune-suppressive potential comparable to that of iTreg cells, which was contributed by reducing IL-17A expression and the enhanced expression of Foxp3, leading to the increase of IL-10 secretion. Consistent with this result, mRNA sequencing analysis of ntHIF-1α-TMD-treated TH17 cells revealed that the expression of the genes involved in TH17-cell functions was downregulated. Also, the genes essential for the immune-suppressive functions were upregulated. The immune-suppressive potential of ntHIF-1α-TMD was further confirmed in an autoimmune mouse model with EAE. Treatment of EAE mice with nt-HIF-1α-TMD effectively alleviated the clinical symptoms and reduced proinflammatory cell infiltration into the spinal cord, probably due to increased CD4+IL-17A-Foxp3+ T cells in the lymph nodes.

In conclusion, we demonstrated that nt-HIF-1α-TMD, the competitive inhibitor of endogenous HIF-1α, could specifically suppress HIF-1α-mediated expression of the genes essential for the functions of TH17 cells, leading to the functional conversion of TH17 cells into immune-suppressive T cells and restoration of immunological balance in the normal physiological condition *in vitro* and *in vivo*. This result is meaningful because therapeutic strategy targeting TH17 cells in autoimmune diseases had been actively studied. For example, secukinumab, anti-IL-17A antibody, was tested in relapsing multiple sclerosis patients (NCT01874340) and has been tested in psoriatic arthritis (NCT04711902) or lupus nephritis (NCT04181762). Based on our data, ntHIF-1α-TMD showed significant alleviation of TH17-associated diseases, which is comparable with anti-IL-17A antibody. Considering secukinumab’s therapeutic potentials, therefore, ntHIF-1α-TMD can be an essential therapeutic reagent for treating various autoimmune diseases in which TH17 cell is a dominant pathogenic cause.

## Data Availability Statement

The original contributions presented in the study are publicly available. These data can be found here: https://www.ncbi.nlm.nih.gov/sra/PRJNA747838.

## Ethics Statement

The animal study was reviewed and approved by the Institutional Animal Care and Use Committee (IACUC) of Yonsei University (South Korea).

## Author Contributions

J-SS was responsible for designing and performing the experiments, data analyses, and manuscript writing. IK was responsible for designing the experiments and performing *in vitro* experiments. J-SM helped in the experiment design and manuscript editing. C-CH helped in the experiment design. M-SC was involved in a primary cell-based assay. SG provided helpful input for the analyses. S-KL designed the study and was responsible for the conception of the study, editing the manuscript, and final approval of the manuscript. All authors contributed to the article and approved the submitted version.

## Funding

This work was supported by a Global Research Laboratory (GRL) Program of the National Research Foundation of Korea (NRF) grant funded by the Korean government (Ministry of Science and ICT) (NRF-2016K1A1A2912755) and Brain Korea 21 (BK21) PLUS Program. J-SS is a fellowship awardee by BK21 PLUS program, South Korea.

## Conflict of Interest

S-KL is employed by Good T Cells, Inc.

The remaining authors declare that the research was conducted in the absence of any commercial or financial relationships that could be construed as a potential conflict of interest.

## Publisher’s Note

All claims expressed in this article are solely those of the authors and do not necessarily represent those of their affiliated organizations, or those of the publisher, the editors and the reviewers. Any product that may be evaluated in this article, or claim that may be made by its manufacturer, is not guaranteed or endorsed by the publisher.
