# Supplementary material for: Intranuclear Delivery of HIF-1α-TMD Alleviates EAE via Functional Conversion of TH17 Cells
Source: Front Immunol. 2021 Oct 21;12:741938. doi: 10.3389/fimmu.2021.741938 (PMC8566938; doi:10.3389/fimmu.2021.741938)
Supplement: Supplementary file 1 [file DataSheet_1.docx]

**Supplementary Material**

## Supplementary Figure 1


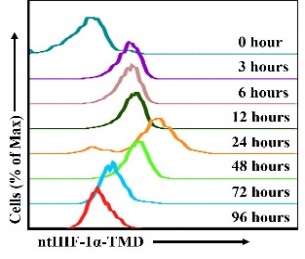

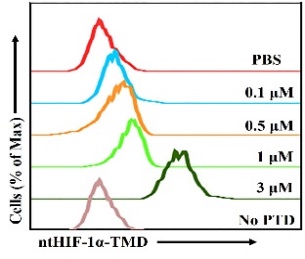

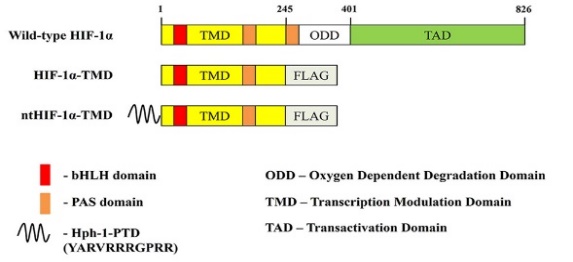

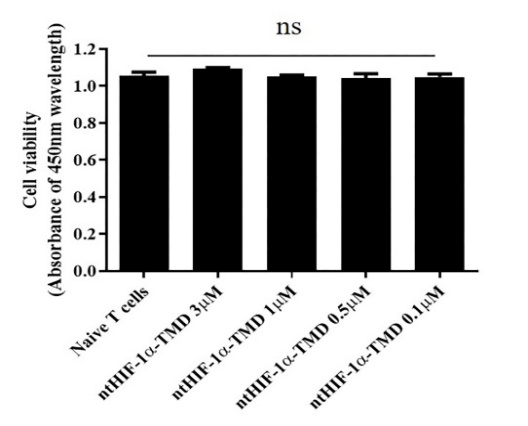

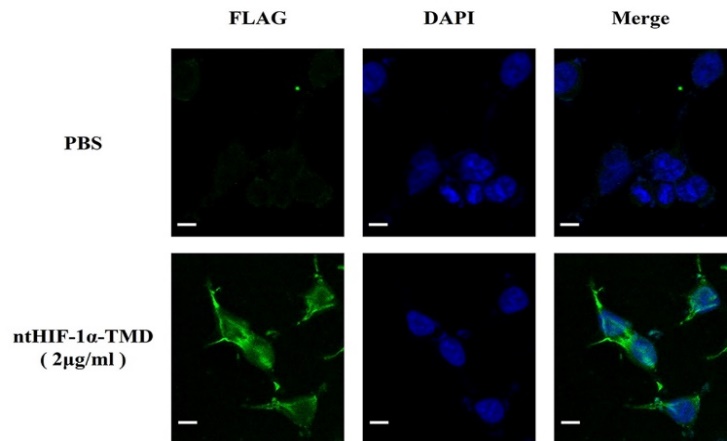

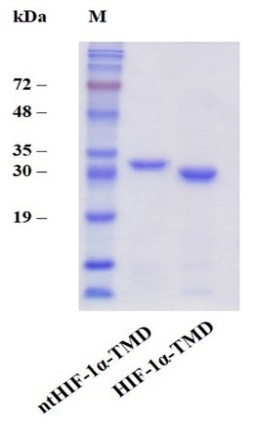


(B)

(A)

(C)

(E)

(F)

(D)

**Supplementary Figure 1.** Generation of ntHIF-1α-TMD and verification of its intra-nuclear localization. (A) Structure of HIF-1α-TMD without protein transduction domain Hph-1 (Hph-1-PTD), and nucleus-transducible form of HIF-1α-TMD (ntHIF-1α-TMD). (B) Purified HIF-1α-TMD and ntHIF-1α-TMD were confirmed for their purity and identity by SDS-PAGE or western blot. M: protein marker. (C, D) Dose- or time-dependent protein delivery kinetics were examined in Jurkat T cells. (E) By confocal microscopy, intranuclear localization was analyzed with an anti-FLAG antibody in HeLa cells. Scale bar = 10μm (F) Cytotoxicity of ntHIF-1α-TMD was evaluated in mouse naïve CD4+ T cells treated with different concentrations of ntHIF-1α-TMD. The experiments were performed at least twice. Data are represented as mean ± SEM (n≥6), and Student’s t-test was used for statistical analysis of group differences. ns; not significant.

##
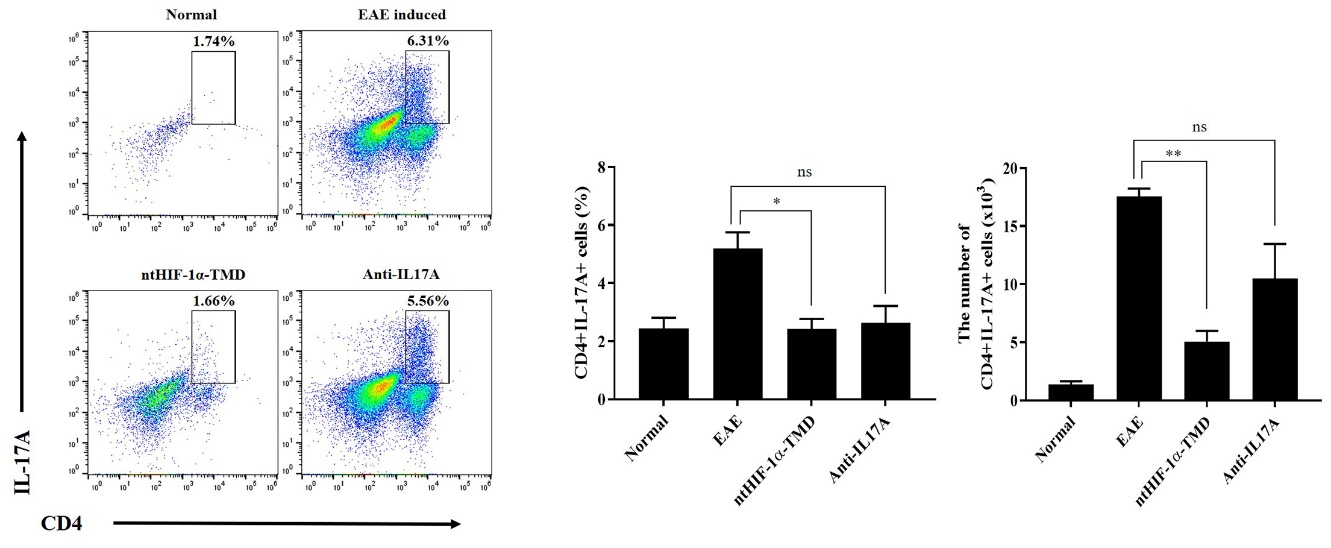

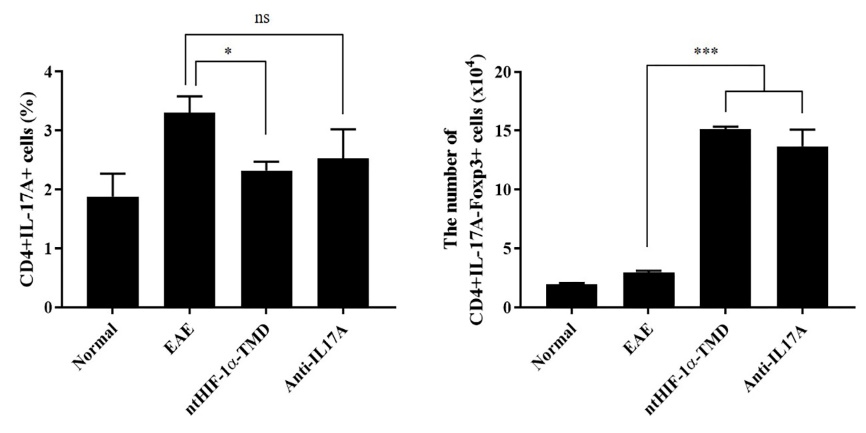
Supplementary Figure 2

(B)

(A)

**Supplementary Figure 2.** Analysis for CD4+ T cell population in EAE mouse model. (A) Flow cytometry analysis was performed to investigate the proportion CD4+IL-17A+ cells or the number of CD4+IL-17A-Foxp3+ cells in the draining lymph nodes. (B) CD4+IL-17A+ cells infiltrating spinal cord were also analyzed. The graphs are represented as mean ± SEM (n≥4), and Student’s t-test was used for statistical analysis. ns; not significant, *P<0.05, **P<0.01, and ***P<0.001.
